# PCD Receives Highest 5-Year Journal Impact Factor in Its History: Celebrating and Building on 20 Years of Success With Release of 6 Collections in 2025

**DOI:** 10.5888/pcd22.250248

**Published:** 2025-07-10

**Authors:** Leonard Jack

**Affiliations:** 1 Preventing Chronic Disease, Office of the Director, National Center for Chronic Disease Prevention and Health Promotion, Centers for Disease Control and Prevention, Atlanta, Georgia

**Figure:**
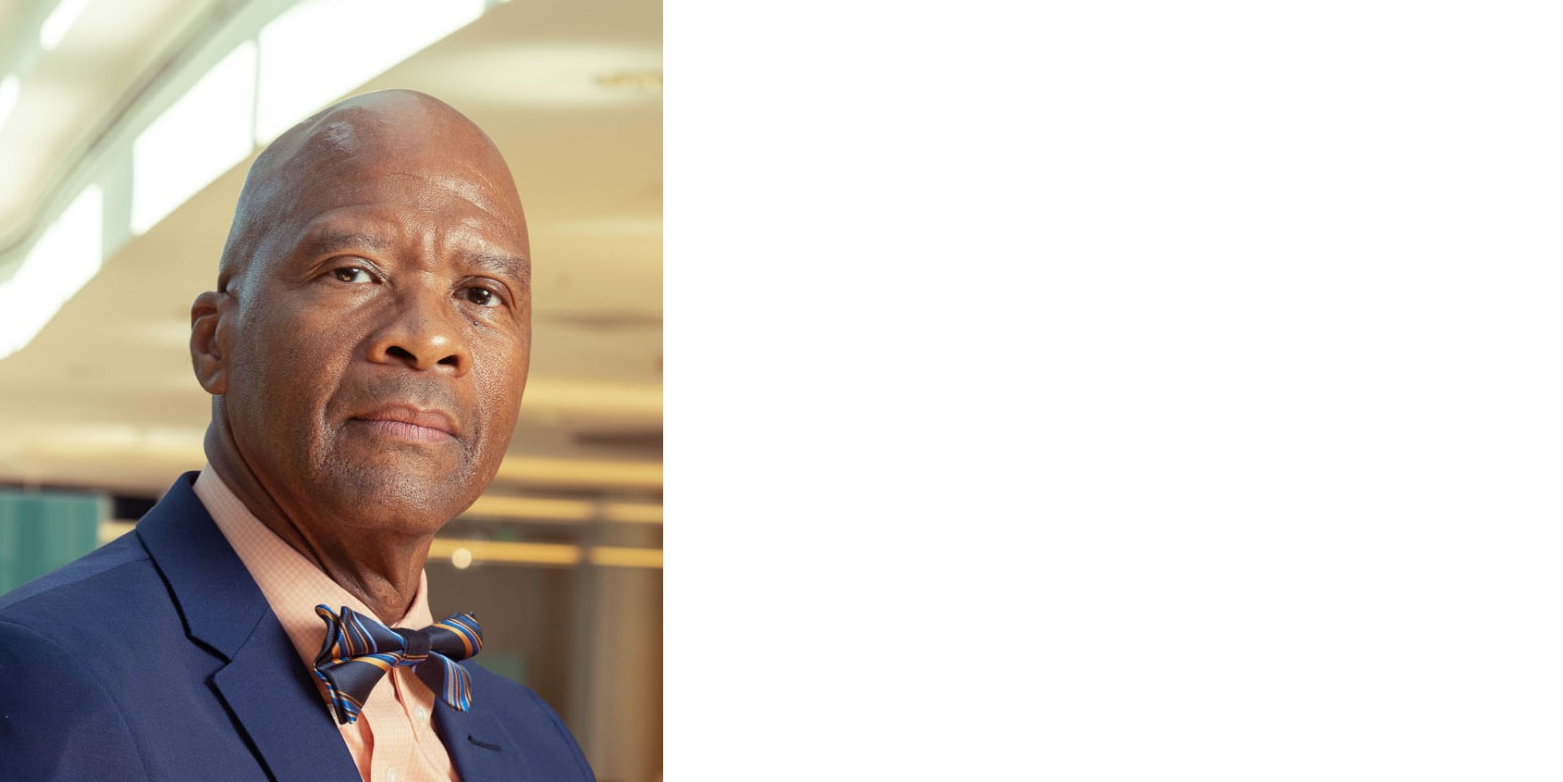
Leonard Jack, Jr, PhD, MSc

In 2024, *Preventing Chronic Disease* (PCD) celebrated 20 years of publishing timely peer-reviewed content generated by subject matter experts from around the world. Year after year, PCD has achieved remarkable accomplishments, and 2025 is no different. PCD announced receipt of a 2024 Journal Impact Factor of 3.9 in the 2025 release of Journal Citation Reports (JCR) in the category of Public, Environmental & Occupational Health. Most impressive is the increase in PCD’s 5-year Journal Impact Factor, from 4.3 for 2023 to 4.6 for 2024. This year’s 5-year Journal Impact Factor is the highest the journal has received since the journal was established in 2003. The 5-year Journal Impact Factor represents the average number of times that an article published in the previous 5 years (2019–2023) was cited in 2024. JCR also ranked PCD in the top 6% (43rd of 699) of journals worldwide in the category Public, Environmental & Occupational Health. Scimago Journal & Country Rank (SJR) ranked PCD third of 33 open-access US public health journals, placing it in the top 6% worldwide of all 31,136 journals and in the top 10% (16th of 167) of US journals in the category Public Health, Environmental & Occupational Health. PCD is indexed in PubMed and PubMed Central and has an acceptance rate of 21.9%.

Teams of dedicated volunteers — serving on the journal’s Editorial Board, Associate Editorial Board, and Statistics Review Committee — along with peer reviewers and journal staff members helped to make the journal a sought-after and well-respected resource for researchers, evaluators, and policymakers worldwide. PCD sincerely thanks its readership for trusting the journal to provide reliable content that has been used to inform public health responses to persistent and novel challenges that require new knowledge and innovation. The journal looks forward to releasing additional rich content on topics of greatest interest to the journal’s readership and the field of public health at large.

Already this year, PCD has released 4 collections that speak to the journal’s commitment to keep its readership up to date on new findings in public health research, evaluation, and practice.

On March 20, PCD released its first collection of 2025, Public Health Research and Program Strategies for Diabetes Prevention and Management. This collection features 13 peer-reviewed articles, plus a peer-reviewed guest editorial, that describe research and evaluation related to identifying barriers to the prevention and management of diabetes and strategies for implementing and evaluating evidence-based approaches aimed at reducing the burden of this disease in the United States.

On June 5, PCD released its second collection of the year*,* Community Engagement and Population Health. This collection of 9 peer-reviewed articles, plus a peer-reviewed guest editorial, focuses on community engagement in public health, from practice to evaluation. These articles describe community engagement strategies, advances in measuring community engagement, and examples of community engagement, partnerships, and collaborations in disease prevention and health promotion.

On June 19, PCD released a third collection, Rural Health Disparities: Contemporary Solutions for Persistent Rural Public Health Challenges. The objective of this collection was to explore factors that contribute to the well-being of individuals in rural areas and multifaceted strategies for public health interventions that aim to decrease the prevalence of chronic disease among rural populations. The 9 peer-reviewed articles in this collection include such topics as nonmedical determinants of health, environmental influences, policy interventions, and community-based initiatives. This collection also includes a peer-reviewed guest editorial.

On July 10, a fourth collection was released: Strong Heart Study: 35 Years of Research Collaboration With American Indian Communities. This collection describes findings from the Strong Heart Study, a 35-year collaboration among investigators, researchers, and American Indian communities aimed to improve the cardiovascular health of American Indian populations. It includes 6 peer-reviewed articles, plus a peer-reviewed guest editorial.

In the coming months, PCD will release 2 additional collections. First, PCD will showcase student authors from across the US. This annual collection features articles submitted by high school, undergraduate, graduate, doctoral, and postdoctoral students addressing a range of public health issues and demonstrates PCD’s commitment to providing scientific writing opportunities to the next generation of public health professionals. Our final collection for 2025 will be Chronic Conditions, Multimorbidity, and Health Outcomes Among US Adults.

PCD’s mission is to promote dialogue among researchers, practitioners, and policymakers worldwide on the integration and application of research findings and practical experience to improve population health. This year’s achievements of reaching PCD’s highest 5-year Journal Impact Factor and maintaining its domestic and worldwide rankings in the sphere of scientific publishing demonstrate that the journal remains a reliable and sought-after resource for guidance in public health research, evaluation, and practice recommendations. Thank you for your continued support of PCD.

